# The meaning of long-term caregiving for patients with frontal lobe dementia

**DOI:** 10.3402/qhw.v8i0.19860

**Published:** 2013-02-20

**Authors:** Hege Rasmussen, Ove Hellzen

**Affiliations:** 1 Department of Psychiatry, Namsos Hospital, Nord-Trøndelag Health Trust, Namsos, Norway; 2 Department of health and Science, Nord-Trøndelag University College, Namsos, Norway

**Keywords:** Frontal lobe dementia (FLD), hermeneutics, insecurity, narrative interviews, nursing care, phenomenological, relation, safety

## Abstract

Nursing staff that work with patients with frontal lobe dementia (FLD) experience challenges that may lead to physical and psychiatric distress. The aim of this study was to capture the feelings, experiences, and reflections of the health staff regarding interactions with and caring for patients with FLD and to highlight what it means for health staff to care for patients with FLD through their daily work. This is a qualitative study with a phenomenological hermeneutic approach. Ten health staff members who work with patients with FLD were interviewed using semistructered interviews. The focus during the interview was the experiences of the staff through their everyday work. The interviews were recorded and then transcribed verbatim. The material was analyzed using a phenomenological hermeneutic approach. The result of the study identifies three themes that highlight the meaning of caregiving for patients with FLD, that is, being aware of the relationship with the patients, being insecure, and being safe. The patients’ unpredictable behaviour puts the relationship between the staff and the patients on trial. It is essential in caregiving to see the human behind the disease and the behaviour. The interest of finding new solutions in the caregiving is awakened through the relation with the patients, through reflections with colleagues, external guidance and by support from the staff leader.

Frontal lobe dementia (FLD) is a number of different diseases that is characterized by dementia involving the frontal lobes (Engedal & Haugen, 2004; Melin & Olsen, 2006). FLD represents an important cause of dementia among late-middle-aged individuals (Melin & Olsen, 2006; Moretti, Torre, Antonello, & Cazzato, 2003). The diseases are spoken of as FLD, frontotemporal degeneration, temporal variant FLD, frontal variant FLD, taupathier, picks dementia, and Frontotemporal lobe dementia (FTLD) (Melin & Olsen, 2006). What is common for the types of dementia that involves the frontal lobes is that the first symptoms are usually changes in personality and changes in behaviour. The early symptoms of FLD can include changes in mood with signs of anxiety, depression, or severe sentimentality (Engedal & Haugen, 2004; Melin & Olsen, 2006) Frontal lobe dementia is characterized by focal atrophy of the frontal and temporal lobes. It encompasses three clinical variants: a behavioural variant, and two language variants (Mioshi, Bristow, Cook, & Hodges, 2009).

The behavioural variant (bv-FLD) is the most common (Garcin et al., 2009). In bv-FLD, the decline of cognitive abilities and the marked behavioural changes are core characteristics.

The behavioural changes can include disinhibition, apathy, emotional blunting and stereotypic behaviour (Mioshi et al., 2009). Changes in personality and social cognition, altered eating patterns, and decline in manners and self care are known with FLD (Engedal & Haugen, 2004; Garcin et al., 2009). Most people associate dementia with poor memory, but the signs of cognitive changes in FLD are reduced attention, repetition of previous actions, solutions incorrectly repeated in new solutions and tasks, reduced ability to choose strategy, reduced ability to find out what is important and less important when it comes to solving tasks. FLD also includes severe language problems. In serious conditions, the spontaneity of language may be missing altogether (Engedal & Haugen, 2004; Kraus, 2001). Symptoms, such as aggression, irritability, restlessness, anxiety, and depression, are common in FLD and is demanding for caregivers (Engedal & Haugen, 2004; Skovdahl et al., 2003; Yeaworth & Burke, 2000). Aggressive behaviour may cause reactions, such as aggression, astonishment, antipathy, insufficiency, powerlessness, insult, and fear, in the caregivers (Åstrøm et al., 2004). Nursing staff that work with patients with FLD experience challenges that may lead to physical and psychiatric distress for health staff (Merrilees & Miller, 2003; Mioshi et al., 2009; Mourik et al., 2004; Yeaworth & Burke, 2000) Some of the difficulties in caring for people with dementia involving predominantly frontal-lobe dysfunction are ethical issues whereby the residents integrity must be balanced against a safe and secure environment (Edberg & Edfors, 2007). Most nurses are familiar with dementia and Alzheimer's disease (AD), but the dementias associated with other neurogenerative diseases are often less familiar. There are important cognitive differences between AD and FLD. Generally, memory is preserved until later in the disease with FLD. Caring for this group of patients is nearly always extremely challenging, yet often rewarding (Merrilees & Miller, 2003). Because FLD often starts early, at 40–65 years of age, the symptoms of the disease have consequences that differ from other dementia diseases. The person affected is often in a productive stage of life (Edberg & Edfors, 2007). There is relatively little literature dealing with patient behaviour, caregiver burden, and possible behavioural interventions in caring for patients with FLD (Edberg & Edfors, 2007; Merrilees & Miller, 2003; Yeaworth & Burke, 2000). Depression and stress in FLD caregivers is higher than in AD caregivers (Mioshi et al., 2009).

The aim of this study was to illuminate the meaning of caring for patients with FLD as experienced by health staff in a Norwegian nursing home.

## Methods

The study is a qualitative study with a phenomenological hermeneutic approach, which has drawn on Ricoeur's (2008) insights into experience; we can never understand another individual's experience, but it is possible to understand the meaning of it. The experience remains private, but its meaning becomes public through interpretation. The method has been developed at the Department of Nursing, Umeå University, Sweden, and the Unit of Nursing Science at the University of Tromsø, Norway (Lindseth & Norberg, 2004).

Human beings live and act out of their morals without necessarily knowing about them and are therefore not usually able to explain their ethical thinking. To gain access to the ethical thinking, one has to ask the health staff to tell stories from situations in their everyday work and interaction with the patients. These stories are captured in narrative interviews (Lindseth & Norberg, 2004). During the narrative, it is possible to shift from the natural to a phenomenological attitude. The natural attitude is an attitude in which we judge and already have made judgements about the phenomena. To shift to the phenomenological phenomena we must accomplish epoche or bracketing. The most natural way of doing this is to have a narrative about lived experience. The listener may not judge but rather participate in the story: “So this is what you have experienced, so this is what you thought.” Then both listener and narrative are free to consider; what are the essential characteristics of the expressed meaning? (Lindseth & Norberg, 2004) The goal in narrative interviewing is to generate detailed accounts rather than brief answers or general statements. One story can lead to another, as narrator and listener or questioner negotiate openings for extended turns and associative shifts of topics. When shifts occur, it is useful to explore, with the participant, associations and meanings that might connect several stories. If we want to learn about an experience in all its complexity, details count (Riessman, 2008).

## Setting and participants

The study was performed in a nursing home in the middle of Norway, at a geriatric psychiatric unit. The sample consisted of 10 staff members who work with patients with FLD, chosen in cooperation with the unit leader. The researcher wanted participants who had worked at the unit for at least one year. The participants had different professions, such as nurse, social educator, licensed practical nurse and social worker. The participants are therefore referred to as health staff in this study. Two participants withdrew and were replaced by two others. At the time of the study, four patients were diagnosed with frontal lobe dementia.

## Data collection

The data were collected through a narrative interview developed for the study on the basis of literature review. The interview guide consisted of five main questions and several underlying themes and sub themes, but the participants were welcome to speak freely about their experiences. A “re-enactment” technique (Drew, 1993) was used by the researcher to investigate the phenomenon, where the informants were encouraged to narrate one or several self-experienced situations in as much details as possible. Each informant was asked to speak (narrate a story) about the following themes: “Could you tell me about a typical day at work, where you are giving care for patients with FLD?”, “What do you find important to have knowledge of when it comes to caregiving for patients with FLD?”, “Can you tell me about a situation at work where you managed to give good care for the patient?”, “Can you tell me about a situation at work where you did not manage to give good care for the patient?”, “Can you tell me about in what matter the patients behaviour affects you?” Using stories as a data collection method is supported by Sandelowski who feels that an individual's story gives important information about the person who narrates (we are the stories we tell) and that a story includes, in itself, the past, the present, and future (Sandelowski, 1994) The interviews lasted for 45–60 minutes and were recorded. The recorded interviews were transcribed verbatim into Norwegian immediately after the interview.

Nine of the staff members were interviewed during their working hours in an office at the nursing home. One staff member was interviewed at home. Two test interviews were carried out initially which resulted in some changes in the questioning.

## Analysis

A phenomenological hermeneutic approach inspired by the philosophy of Ricoeur (2008) was chosen to illuminate the staff members’ lived experience of caregiving for patients with FLD. According to Ricoeur (2008), the interpretation process involves three phases: a naive reading, a structural analysis, and a comprehensive understanding. The purpose of the structural analysis is to explain parts of the text and validate or invalidate the understanding obtained from the naïve reading. The analysis started with a naïve reading of the text: the text was read as a whole repeatedly to get an understanding of the whole material in relation to the study question. The text was then analysed by structural analyses. The text was read several times and sorted into meaning units which could either include some words in a sentence or several sentences bound together by their content, guided by the aim of the study and the naïve understanding of the text. The meaning units were then coded, condensed, and grouped into sub-themes. The next step was to group the sub-themes into themes. A summary of the analyses can be seen in Table I.

**Table I:** Summary of the stuctural analyses

Meaning units from transcribed text	Condensed meaning units	Sub-theme	Theme
To see that the human being actually is still there. You get in touch with the human behind the disease in a way and you get more go-ahead spirit.	A lucid moment in the patient gives an opportunity to have an equal conversation with the patient and to achieve a feeling of having done a good job.	Being close to the patient.	Being aware of the relation with the patient.
Sometimes the patients see themselves and their situation. Then you can tell from their expression that they are devastated.	Sympathy and empathy with the patients arises when the patients realize their own situation.	Being close to the patient.	
We should not keep what has been against the patient, but we should not forget it either.	The difficult period with severe aggressive behaviour is important to remember.	Being scared of the present situation getting worse.	Being insecure.
Doing something you really do not want to do is worse. You feel like you are crossing some limits.	Doing something against the patients will gives a feeling of not giving good care for the patient.	Being uncertain about caregiving.	
When they are able to be relaxed and you get the good moments.	To observe relaxed non-verbal language in the patients feels good and satisfying.	Being aware of the patient having a good moment.	Being safe.
It is important to have knowledge regarding that the patient's behaviour is not about being evil but about having a disease.	To know the person and to know the disease gives a feeling of being able to handle the patient in a better way.	Being able to be a step ahead and handle aggressive behaviour in a professional way.	

In the final step, the text was again interpreted as a whole in light of understandings achieved from naïve reading, the analysis, and the researchers’ pre-understanding. A comprehensive understanding was then made by the authors. Finally, identified patterns or processes were considered in relation to existing research and theories (Lindseth & Norberg, 2004).

## Ethical review

Permission to carry out the research was granted by the Norwegian Social Science Data Services (NSD) 2.2010/23573. Data collection followed the guidelines of the Data Inspectorate of Norway. The staff and their unit leader received written information of the study, including a request for participants. The researcher received an invitation from the unit and gave verbal information about the study at a unit meeting. The staff received information about the purpose of the study, their ability to retire from the study at any time, the confidentiality of the study, and the use of information gained from the interviews. Given that the interviews might evoke feelings of discomfort concerning situations experienced, all interviewees were told they were welcome to contact the researcher or the unit leader if they wanted to discuss something related to the interviews, though no one took advantage of that opportunity. Every participant signed a consent form before the interviews.

## Findings

### Naïve reading

The staff members’ everyday work is unpredictable because of several changes in the patient's physical and psychiatric condition during the day.

The unpredictability is handled by the staff by being observant through reading the patients verbal and non-verbal language. To be able to do this, they use their knowledge of the patient and their intuition. To work with experienced colleagues who understand the patients and who are capable of handling difficult situations with patients is important to the staff members to be safe. In some cases, a special chemistry is achieved between staff members, where they are able to communicate non-verbally in situations with the patients. The staff members try to avoid anxiety, stress, and aggression in the patients by being a step ahead of the patients using their observations, and by having calm body language. Aggressive behaviour from patients may lead to difficult feelings towards the patients, such as anger. This creates insecurity as to whether the caregiving is ethically and morally legal. Knowledge of the diagnosis and the person behind the diagnosis is important when it comes to being able to handle aggressive behaviour from the patients. It is important for the staff members to experience good moments with the patients. This is described as a motivator for the everyday work. During these moments, it is possible to connect with the patient and improve the relationship. This also leads to feelings of empathy and sympathy with the patients and being aware of the patient's vulnerability in his or her situation. The unit leader and the working environment are important for the staff members. The feeling of being seen and heard by the leader and the colleagues is described as important. This, including external supervision, has given the staff members better confidence in the caregiving.

## Structural analyses

### Being aware of the relationship with the patients

The staff members find the caring complicated because of the patients’ difficulties in expressing their needs. The patients also often resist what staff members see as important in caring. In these situations, it is seen as crucial to have a relationship with the patient. An important part of creating a relationship is usually communication, which is often challenging for patients with FLD. Observing and understanding the patient's body language is described as important have an understanding of the patient's wellbeing. As the social educator describes:

I notice body language, things they say and do … I don't think this is something you could just learn, you can't sit still on the school bench and learn this, you have to know the person in question because everybody is different.

The staff members tell of an important arena of building a relationship with the patients, the so-called “clear moments.” Clear moments are described as short moments where the patients are able to be present during the conversation and can seem fully oriented. These moments create joy and astonishment among the staff members and a feeling of the patients achieving life quality arises. The staff members use these moments to get to know the patients better. One of the interviewed licensed practical nurses narrates:

Sometimes when you sit with her she can connect and talk about what she is thinking about … it feels good being able to have a conversation. It is like you are able to see the woman beneath.

During the clear moments the patients can talk about themselves, their family, and experiences in life. An equal conversation appears between patient and staff members sharing personal experiences. The staff members describe this as a motivator to keep working. At the same time, it is difficult to avoid becoming too personal in that the patient becomes too attached and too dependent. The staff members talk about he patients that are not willing to let all the staff members get close to them, but also that some staff members seem to have a better chemistry with different patients. In this case, some staff members are able to communicate through use of humour with the patients.

Clear moments in the patients give a sense of having done a good job, a feeling of joy, desire of leaving bad moments behind, and desire to experience more clear moments. It feels good to have an equal conversation where respect for the patient and maintenance of the patient's integrity is possible. As a nurse describes:

The clear moments mean a lot. I believe that if we didn't experience them, it would be dispiriting working here. It means a lot that we have these glimpses of light.

To know the human and to build a relationship with the patient also involves the staff getting closer to the patients’ suffering. Sometimes, during a clear moment, the patients experience hopelessness in their own situation. This is a strong, devastating and difficult experience for the staff members. They feel empathy and sympathy and can also feel despair and sadness about the situation. The patient can also become tired and anxious after clear moments.

In this way, the clear moments may also be stressful for the patients and for the staff.

The staff members also find knowledge of the disease as important to maintain the relationship with the patient. This is to separate between personal qualities and symptoms of the diagnosis. A social educator mentions:

It is important to know that inappropriate behaviour is not evil but it comes from the disease, because I have seen many who have … it is ok to feel angry. But there are many who say stuff like: behave yourself!!.

The patients challenging behaviour creates the experience of being humiliated and rejected. This leads to feelings of reservation, anger, powerlessness, fear, and thoughts of yelling at or hitting the patient. This puts the relationship between the patient and the staff members on trial. These feelings cause psychological and physical tiredness, guilt towards the patient, sadness, and a feeling of having done a bad job. The balance between meeting the patients’ needs and setting boundaries feels difficult. It is important to have a relationship with the patient to understand how the patient is doing, what kind of needs the patient has, and to predict the patient's actions. In this way, anxiety in the patient may be prevented. Staff members describe that the experience of working with a patient with FLD cannot be transferred to other patients with the same diagnosis. A social educator describes:

I know the patients so well; I soon recognise what is about to happen because I know them. But when we meet new patients, you can't transfer this; you have to get to know the patient.

### Being insecure

Sometimes the staff members went through longer periods of aggressive behaviour from the patients. In some cases, the staff members had to use force to restrain patients from hurting themselves and others. At first the FLD diagnosis and the symptoms were unknown to the staff members. The staff members did not find much literature describing caregiving for patients with FLD. The demanding situation made it difficult for the staff to update their knowledge. Some of the staff members were physically injured because of the patients’ aggressive behaviour. Some went on sick leave for a longer period of time. Staff members talk about this period being filled with despair, fear, powerlessness, stress, and tiredness. A social worker describes:

We went to work expecting that today we will get beaten up. But we pushed on through it and … we cried on each others shoulders and expressed it in the unit office, but the situation was horrible.

The situation created ethical dilemmas in caregiving and insecurity regarding having done a good job. The caregiving often consisted of trying to stop aggressive behaviour and to prevent someone getting hurt. It felt wrong and brutal to restrain a patient against his will and to prevent aggressive behaviour. As a social educator narrates:

It makes you feel like you are crossing some limits … you are supposed to give care not to hold the patient … I can stand the aggressive behaviour but having to do something I really don't want to do is worse …

The staff members tell us about insecurity when it comes to caring, because this way of caring was not equal to the usual and known type of caring. Not only was the staff physically injured by the patient through biting, kicking, hitting, pinching, and hair pulling but they also experienced verbal harassment, spitting, and being thrown at with faeces. The situation created negative thoughts and feelings towards the patient, and this created insecurity when it came to holding the patient harder then necessary. This created insecurity to whether the patient's treatment was ethical and morally correct. The staff members tell about despair regarding the local government legal system when it came to using force against patients. A nurse describes this:

I thought … I was really upset because it wasn't possible to get an allowance to use straps on the patient. I would have been better for the patient and better for us.

According to this legal system, a patient cannot be kept against his or her will, unless it is an emergency. The staff members were instructed to document every situation where force had been used, and some were concerned that what they did to the patient was illegal and that they could loose their job. Many staff members would have liked to use physical aid-like straps, but the legal system did not allow this. The situation was devastating and the need for external guidance emerged. Situations with severe aggressive behaviour also created insecurity when it came to managing the situation, so that no one was injured. The staff members became uncertain of which of the colleagues would be able to handle the situation. It became important that staff members had an open dialogue regarding if they did not feel able to enter situations and to share thoughts and experiences with colleagues. As a licensed practical nurse narrates:

To talk about things with my colleagues, let it out and try to get awareness of how I interact with the patient, in this way I have also learned a lot about myself during these years too …

Some of the insecurity that the staff members describe in their everyday work at the time of the interviews emerges from this earlier period. This shows as concern among the staff regarding if a new period with severe aggressive behaviour would emerge. To avoid stressing the patients, the staff members are aware of their own verbal and non-verbal language. The staff members are striving to get to know the patients, to read their verbal and non-verbal language, and balance closeness and distance to avoid stressing the patients. This balance is described as a demanding and tiresome work method.

The insecurity comes also from the patient being unpredictable, which can lead to difficulties planning work. The patient can, for example, resist taking showers even if the patient's condition requires this. The staff members are torn between considering the patient's needs or physically caring for the patient's hygiene, in spite of the knowledge that this could lead to aggressive behaviour. When planning is not possible, the staff members choose to seize the moment when the patients seem most receptive. The staff members also know that the patients may become tired and stressed after, for example, showering, activities, or having visitors.

### Being safe

A relationship with patients represents being safe, because observation of the patient gives information about how he or she is doing and what he or she needs. As a nurse describes:

When I get to work I try and see what condition the patient is in. I can tell by positure … mood … the staff can tell what condition the patient is in without speaking to each other.

The relationship between colleagues is also important for the staff members when it comes to being safe. Working with a colleague who knows the patient is important.

Being in a smaller group that knows each other, the patient, and the routine is seen as an advantage. A special chemistry between colleagues where verbal communication is nearly unnecessary to understand each other is described. A licensed practical nurse describes this chemistry as being coordinated:

Being coordinated means that we … together … can predict actions that may occur. We kind of see the situation together; we all know what to do without speaking to each other. We are a step ahead, the intuition is constant.

The patients are described as sensitive when it comes to the staff members’ verbal and non-verbal language and also when it comes to communication between staff members. A calm attitude that is not affected by the patients’ distress is important to avoid accelerating distress among the patients. It is important that the staff have an equal understanding that planned caregiving may not always be completed, for example, concerning personal hygiene. The staff members have reflected that this does not mean that they have done a bad job. A nurse narrates:

At first I thought it was hard not be able to finish the tasks I had planned. I felt inadequate. We often use our staff meetings to reflect on these kinds of things.

Through staff meetings, the staff members have achieved an equal understanding of the caregiving situation. Staff members have also received external guidance when it comes to working with aggressive patients. A social worker describes the importance of this:

Through the guidance we have learned that it's not our fault when patients get aggressive. It is not personal; it is an expression of frustration. I was very grateful for the guidance. Someone from outside came and saw our situation.

Several staff members had worked at the unit for many years and have participated in development of competence when it comes to patients with FLD, through their own experience, reflection, and guidance. The staff members describe their unit leader as important for their every day work, in that they feel seen, heard, and taken care of by their unit leader. The unit leader also motivates staff to reflection and new initiative. The unit has also received guidance from a special unit in Denmark with patients with FLD. The unit has appointed a staff member that has responsibility for the development of competence. This ensures that the caregiving staff members are professional. The staff group has an identity and is proud of having this competence.

## Comprehensive understanding

The interpretation suggests that caregiving for patients with frontal lobe dementia means giving care to patients who are unpredictable and vulnerable. The staff members are required to give a different kind of care which is about being close to and curious about the person and being flexible to meet the person where he or she is in the actual moment. This is a demanding balance, as getting too close to the patient could result in distress for the patient. The staff members use their experiences, observations, and intuition as tools in this balance. It seems like each staff member knows what it means for the other staff members to give care for the patients and that this sometimes results in a special chemistry between the colleagues. This chemistry is also based on the relation to the patient. This chemistry allows the staff members to communicate using non-verbal language. This is described as a useful tool in caregiving because the atmosphere around the patients remains calm and quiet. Aggressive or challenging behaviour from patients leads to difficult feelings, but the knowledge of the person behind the disease and the disease itself makes the staff members see the aggression as an expression of frustration. The staff members are vulnerable in the setting of trial and error in the caregiving, but the interest of the patients and the interest in developing competence keeps them going on in their everyday work.

## Discussion

Findings in this study suggests that caregiving for patients with FLD means being aware of the relationship with the patient, being insecure, and being safe.

The staff members find it important to get to know the person behind the disease to give good care but find this challenging because of the symptoms of the disease. It is well-known that the relationship with patients that insures dialogue and communication is crucial to understand and explain the patient's reality (Lingsås, 2008).

As the patients have severe challenges in verbal communication, the staff members take advantage of the patient's clear moments to learn about the person behind the disease. To experience episodes of lucidity is seen as important among the staff members, especially in the case of achieving a relationship with the patient. During clear moments, an equal conversation can arise between patient and staff and information about each other's lives are shared. Patients with dementia can experience episodes of psychiatric clearness. This can be explained by episodes where patients with dementia have shown clarity through words and actions that unexpectedly and surprisingly appear. These moments come surprisingly when there is no demand on the patient, and they are called episodes of lucidity (Normann, Asplund, & Norberg, 1998). The staff members describe this as a moment where the patient is calm and seems content and then suddenly is able to “connect”.

Building a relationship is also seen by the staff members as challenging because of the difficult balance between getting too personal with the patient and getting too detached from the patient. Edberg and Edfors (2007) find in their study that occasionally it is hard for the staff to set a boundary between being personal and professional. The relationship can sometimes be misread by the patients and they can become dependent or feel in love with a staff member (Edberg & Edfors, 2007). Patients with FLD are often younger which could complicate the relations. The patient could be at the same stage of life as the staff member, with younger children and grandchildren (Garcin et al., 2009).

The staff members describe that when the care of the patients includes restraining because of inappropriate or aggressive behaviour, it puts the relation between staff members and patients on trial, because this behaviour leads to negative thoughts and feelings about the patient. Restraining the patients also leads to insecurity regarding having caused them some harm.

Fagerberg and Engstrøm (2012) find in their study that care of the old is a matter of ethics, organization, and relationships. Ethical moral self is one of the three themes in their study, with the sub-themes “pliability towards the old” and “difficulties meeting the aggressiveness”.

The ethical moral thinking is challenged when caring for very ill older persons with aggressive behaviour (Fagerberg & Engstrøm, 2012). This is in line with Eriksson and Saveman (2002) who find in their study that disorderly conduct in patients suffering from dementia gives rise to ethical conflicts in the caregivers, which can lead to feelings of hatred towards the patients and themselves.

To understand the patients’ non-verbal language and behaviour, the staff members use empathy and fantasy. The staff members are trying to maintain curiosity in the patient to give care and at the same time balance between getting too close and getting too detached. A person-centred approach is one of several management strategies used when it comes to aggressive behaviour by people with dementia in residential care settings, where attempting to understand the poorly communicated need expressed by the aggressive person is focus. The person-centred approaches are held to be ethical and acknowledge aggressive behaviour as a reaction to events in the person's environment, including the occasional un-therapeutic approach adopted by professional carers (Pulsford & Duxbury, 2006).

The staff members do not describe or name their approach as person-centred, but the elements of this kind of management is present in their approach. They find it crucial to remain curious about the person to be able to remain in their everyday work.

This is in line with the finding in Edberg and Edfors study (2007) where the nurses’ experience of possibilities in caring for patients with FLD is mainly related to their own reactions, a result of many years of “trial and error”. Caregivers who strive to understand the meaning behind a resident's behaviour might be much more successful at curbing distressing behaviour than caregivers who act merely in a custodial role (Skovdahl, Kihlgren, & Kihlgren, 2003). They see the person, let themselves move and start seeking alternative ways of caring. Staff members that are focused on the person behind the disease show understanding for the situation and are motivated to increase the possibilities of the patients well-being (Normann et al., 1998).

Some staff members feel uncomfortable being close to the patient because of an earlier period of severe aggressive behaviour. Severe aggressive behaviour is not current in their everyday work at the moment, but the patients’ unpredictable behaviour reminds them that such a situation could happen again. Mourik et al. (2004) investigated the connection between behavioural symptoms in patients with FTLD and stress among the staff. This study showed that agitation or psychosis and mood created stress for staff members (Mourik et al., 2004).

Hummelvoll and Karlsson (2008) claim that once a patient has been labelled as aggressive, it takes time before the behaviour is forgotten even though it happened a long time ago and the behaviour is clearly better.

During an earlier period of severe aggressive behaviour, the staff members became uncertain in the case of caregiving, which at that time often consisted of restrain and force against the patient to avoid injuries. This kind of caregiving conflicted with the usual kind of caregiving, which normally focuses on the patient's integrity and dignity. Working with aggressive patients requires integration between two conflicting ethical principals: ethics of care and recognition ethics. In recognition ethics autonomy is important. It means that patients are equally justified and that patients’ options should be respected. Freedom, independence, and integrity lead to concepts such as freedom of choice, service, and rights. Care ethics takes effect when the patients are helpless, incompetent, and not able to organize themselves. Here, focus is on respect for the patient's handicap rather than respect for co-determination. These two conflicting ethical principals can be reconciled by both principals working for the patient to preserve their dignity (Gulmann, 2001). Caregiving can be seen as giving a gift and what characterizes this is that it is spontaneous, unmotivated, and unconditional. Mercy, sympathy, and trust are necessary in caregiving (Martinsen, 1989). Caregiving can be seen as an ethical activity where all human beings have the same rights of getting good caregiving regardless of social background, somatic, or psychiatric diagnosis. The caregiving can be seen as a gift offered to the patient that the patient gratefully accepts. This is not always the case, for example, when the patient has an aggressive behaviour. This may lead to resignation in the staff; they may have problems acting professionally in the caregiving (Hellzen, 2000).

The staff members tell about ethical dilemmas in their everyday work regarding choosing between ethics of care or recognition ethics, for example, when it comes to hygiene. They feel that ethics of care requires an understanding of the patient's situation and the human behind the disease. The staff members must rely on themselves as tools in the caregiving, where being a step ahead and reading the patients verbal and non-verbal language is important to avoid difficult situations. This is in line with the study of Edberg and Edfors (2007). Edberg and Edfors carried out a qualitative study in 2007 to investigate the staff's experience of difficulties and possibilities in working with patients with FTLD. The difficulties were explained as related to the patient's inhibition and judgement, anxiety, agitation, reduced abilities to express physical needs, egocentricity, unbalanced rest and activity, and depressed mood. The possibilities were explained as related to being a step ahead, being flexible and calm, to create a positive atmosphere, to be trustful, and to do things together.

Being aware of the relationship with the patient and being insecure is connected with being safe.

A relation gives the opportunity of insight of the patients needs. Knowledge of the patient's non-verbal language gives the opportunity to be a step ahead to avoid or handle aggressive behaviour in a professional way.

Reflection and support from colleagues and leaders and external guidance leads to assurance that the way of caregiving is ethical and moral professional.

The staff members tell of a professional development through trial and error in the caregiving, reflection, and guidance where they have come to an understanding of the caregiving.

This is in line with Edberg and Edfors (2007) who found in their study that receiving continuous feedback and support was reported as crucial for the nurses to be able to continue their work in caring for patients with FLD.

The staff members also speak of a special chemistry between colleagues based on working with each other for a longer time and common knowledge and experience of the patient. This chemistry is spoken of as an important tool, which makes the staff members able to communicate with each other non-verbally during the interaction with the patient. This can provide a calm setting with the patient, where seizing the moment when it comes to caring (for example hygiene) becomes possible. Skovdahl et al. (2003) found that staff members working with aggressive residents with dementia felt that competence, solidarity, and support were needed from colleagues to perform their duties (Skovdahl et al., 2003).

## Conclusion

The overall theme seems to be that the meaning of caregiving for patients with FLD is to build relations with patients who are unpredictable and have poor abilities in communication.

A working tool described as “special chemistry” or “being coordinated” in the caregiving for the FLD patients has developed among staff during periods of severe aggressive behaviour. The intentional function for this working tool was to use less verbal and observable communication to avoid anxiety or stress in the patients, and thereby aggressive behaviour. The feeling of safety achieved by being coordinated or having a special chemistry with colleagues seems crucial to be close to building a relationship with the patient even when aggressive behaviours have not been present for a long time.

## Methodological consideration

One threat to credibility is the way the data is collected. All of the staff members who participated in the study work at the same unit, and there is a risk that they, during their daily discussions, influenced each others narratives. However, each narrative was rich in individual details. The narrative interview technique using re-enactment seems to be useful when the attempts are made to capture individual experiences and reflections. The analysis and interpretations have been judged with the authors pre-understanding taken into account (*cf* Ricoeur, 2008). This interpretation is only one of several possible ones. This study illuminates the meaning of caregiving for patients with frontal dementia in a long-time care unit and can be used for development of competence regarding caregiving for this particular group. The special chemistry between staff members is described as an important tool in their everyday work and could be explored in further studies.

## References

[CIT0001] Åstrøm S, Karlsson S, Sandvie Å, Bucht G, Eisemann M, Norberg A (2004). Staffs experience of and the management of violent incidents in elderly care. Nordic College of Caring Sciences.

[CIT0002] Drew N (1993). Reenactment interviewing: A methodology for phenomenological research. Image – The Journal of Nursing Scholarship.

[CIT0003] Edberg A. K, Edfors E (2007). Nursing care for people with frontal lobe dementia: Difficulties and possibilities. International Psycho Geriatrics.

[CIT0004] Engedal K, Haugen P. K (2004). Lærebok demens- fakta og utfordringer.

[CIT0005] Eriksson C, Saveman B-I (2002). Nurses experiences of abusive/non-abusive caring for demented patients in acute care settings. Scandinavian Journal of Caring Sciences.

[CIT0006] Fagerberg I, Engstrøm G (2012). Care of the old - A matter of ethics, organization and relationships. International Journal of Qualitative Studies on Health Well-being.

[CIT0007] Garcin B, Lillo P, Hornberger M, Piguet O, Dawson K, Nestor P. J (2009). Determinants of survival in behavioural variant of Frontotemporal dementia. Neurology.

[CIT0008] Gulmann N. C (2001). Praktisk gerontopsykiatri.

[CIT0009] Hellzen O (2000). The meaning of being a carer for people with mental illness and provoking actions: Carer's exposure in problematic care situations.

[CIT0010] Hummelvoll J. K, Karlsson B (2008). Se mennesket: om forskning og klinisk arbeid I psykiatrisk sykepleie.

[CIT0011] Kraus M (2001). Ikke medikamentell behandling av Frontotemporal demens. Demens.

[CIT0012] Lindseth A, Norberg A (2004). A phenomenological method for researching lived experience. Nordic College of Caring Sciences.

[CIT0013] Lingsås L. G (2008). Etikk og verdivalg I helse og sosialfag.

[CIT0014] Martinsen K (1989). Omsorg, sykepleie og medisin. Historisk filosofiske essays.

[CIT0015] Melin E, Olsen R. B (2006). Frontaldemens- en håndbok.

[CIT0016] Merrilees J. J, Miller B. L (2003). Long-term care of patients with Fronto Temporal Dementia. Journal of the American Medical Directors Association.

[CIT0017] Mioshi E, Bristow M, Cook R, Hodges J. R (2009). Factors underlying caregiver stress in Frontotemporal Dementia and Alzheimer's disease. Dementia and Geriatric Cognitive Disorders.

[CIT0018] Moretti R, Torre P, Antonello R. M, Cazzato A. B (2003). Frontotemporal Dementia: Paroxetin as a possible treatment of behaviour symptoms. European Neurology.

[CIT0019] Mourik J. C, Rosso S. M, Niermeijer M. F, Duivenvoorden H. J, van Swieten J. C, Tibben A (2004). Frontotemporal dementia: Behavioural symptoms and caregiver distress. Dementia and Geriatric Cognitive Disorders.

[CIT0020] Normann H. K, Asplund K, Norberg A (1998). Episodes of lucidity in people with severe dementia as narrated by formal carers. Journal of Advanced Nursing.

[CIT0021] Pulsford D, Duxbury J (2006). Aggressive behaviour by people with dementia in residential care settings: A review. Journal of Psychiatric and Mental Health Nursing.

[CIT0022] Ricoeur P (2008). From text to action: Essays in hermeneutics.

[CIT0023] Riessman C. K (2008). Narrative methods for the human sciences.

[CIT0024] Sandelowski M (1994). We are the stories we tell: Narrative knowing in nursing practise. Journal of Holistic Nursing.

[CIT0025] Skovdahl K, Kihlgren A. L, Kihlgren M (2003). Different attitudes when handling aggressive behaviour in dementia- narratives from two caregiver groups. Aging and Mental Health.

[CIT0026] Yeaworth R. C, Burke W. J (2000). Frontotemporal Dementia: A different kind of dementia. Archives of Psychiatric Nursing.

